# Delay-Aware Energy-Efficient Routing towards a Path-Fixed Mobile Sink in Industrial Wireless Sensor Networks

**DOI:** 10.3390/s18030899

**Published:** 2018-03-18

**Authors:** Shaobo Wu, Wusheng Chou, Jianwei Niu, Mohsen Guizani

**Affiliations:** 1 State Key Laboratory of Virtual Reality Technology and Systems, Beihang University, Beijing 100191, China; wushaobo1990@126.com (S.W.); wschou@buaa.edu.cn (W.C.); 2 Department of Electrical and Computer Engineering, University of Idaho, Moscow, ID 83844, USA; mguizani@ieee.org

**Keywords:** wireless sensor networks, delay-aware routing, energy efficiency, fixed path, mobile sink

## Abstract

Wireless sensor networks (WSNs) involve more mobile elements with their widespread development in industries. Exploiting mobility present in WSNs for data collection can effectively improve the network performance. However, when the sink (i.e., data collector) path is fixed and the movement is uncontrollable, existing schemes fail to guarantee delay requirements while achieving high energy efficiency. This paper proposes a delay-aware energy-efficient routing algorithm for WSNs with a path-fixed mobile sink, named DERM, which can strike a desirable balance between the delivery latency and energy conservation. We characterize the object of DERM as realizing the energy-optimal anycast to time-varying destination regions, and introduce a location-based forwarding technique tailored for this problem. To reduce the control overhead, a lightweight sink location calibration method is devised, which cooperates with the rough estimation based on the mobility pattern to determine the sink location. We also design a fault-tolerant mechanism called track routing to tackle location errors for ensuring reliable and on-time data delivery. We comprehensively evaluate DERM by comparing it with two canonical routing schemes and a baseline solution presented in this work. Extensive evaluation results demonstrate that DERM can provide considerable energy savings while meeting the delay constraint and maintaining a high delivery ratio.

## 1. Introduction

Wireless Sensor Networks (WSNs) have drawn intensive attention from the industrial community recently due to their flexibility, low cost, and powerful networking ability [1,2,3]. They could be applied for long-term surveillance, smart manufacturing, process control, and so on [4,5,6]. In industries, mobile elements are extensively involved in WSNs and play an increasingly important role [7]. On one hand, inspectors or patrol robots would be required to collect sensing data from various deployed industrial sensors (e.g., radiation sensor, gas sensor, temperature sensor) for decision-making. On the other hand, exploiting mobility of mobile entities in WSNs could improve the energy efficiency, load balance and network connectivity. Consequently, data collection in WSNs with mobile sinks (also referred to as mobile base stations or mobile data collectors) has become a significant issue [8].

There exist three sink mobility patterns [9]: (1) *random mobility* where the sink roams in the sensing field randomly [10,11,12,13,14]; (2) *path-controllable mobility* where the trajectory and the sink speed can be controlled to improve the network performance [15,16,17,18,19,20,21,22,23,24,25,26]; (3) *path-fixed mobility* where the sink moves on a fixed path and under strict constraint its motion parameters such as the speed and pause time are also uncontrollable [27,28,29,30,31,32,33,34,35,36].

In the past several years, extensive research has been conducted on efficient data delivery in WSNs with above sink mobility patterns and could be classified into three categories from the perspective of the routing scheme [21]: *single-hop routing*, *immediate multi-hop routing*, and *rendezvous-based routing*. In the single-hop routing, mobile sinks visit each sensor node and gather data via one-hop communication, resulting in low energy cost but extremely high delivery latency [10,17,27,28,29,30]. With respect to the immediate multi-hop routing, all nodes immediately deliver packets to the mobile sink along dynamic routes with multiple hops, enabling real-time data collection at the cost of tremendous energy consumption [11,12,18,19,20,31]. Rendezvous-based routing strikes a balance between aforementioned two methods, wherein data packets originated from deployed nodes will first be transferred to specified rendezvous nodes and then uploaded to the sink when it arrives [13,16,21,22,23,24,25,32,33].

This paper focuses on delay-constrained energy-efficient data delivery towards a path-fixed mobile sink. This mobility pattern is common in industrial scenarios due to restrictions on accessible areas and the requirements of performing assigned tasks (e.g., robots making regular inspections, people walking along roads, and transport vehicles). Several works adopting the rendezvous-based scheme have been proposed for the path-fixed mobility [32,33]. However, they are dedicated to improving the network throughput or energy efficiency while neglecting the delay requirement. Actually, unlike in the situation that the sink mobility can be controlled, the traditional rendezvous-based routing cannot guarantee the delivery delay when the sink path and speed are all fixed. For example, a vehicle may take 2 h for a round trip, incurring high data collection delay, which is not acceptable for many applications. On the contrary, the immediate multi-hop routing cannot utilize the sink mobility for energy conservation. In real life, there are many types of sensing data that have different delay requirements varying in different conditions. For instance, the smoke concentration in an industrial park might be reported every 2 min in normal circumstances but 1 s in emergency situations. How can various delay constraints be satisfied as well as achieving high energy efficiency in this new context?

**Figure 1:**
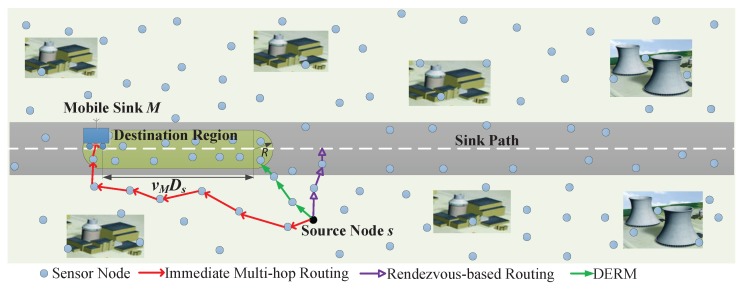
Three routing schemes in wireless sensor networks with a path-fixed mobile sink.

To address this problem, in this work, we propose a Delay-aware Energy-efficient Routing algorithm for WSNs with a path-fixed and uncontrollable Mobile sink (DERM). Figure 1 illustrates the three distinct data routing methods. DERM enables each node to transmit packets to a dynamic region accessible to the sink within the delay constraint (called *destination region*) via the shortest path, and then the packets will be collected before their deadlines when the sink arrives. It can be observed that, compared to the other two schemes, DERM achieves a flexible balance between the data delivery delay and energy consumption. The design of DERM is nontrivial as it intrinsically contains three issues: (1) energy-optimal routing towards time-varying regions; (2) efficient sink location estimation; and (3) a reliable fault-tolerant routing mechanism for handling the location errors. Our major contributions are summarized as follows:

1. To the best of our knowledge, DERM is the first work concerned about both the delivery delay and energy efficiency for data collection in WSNs with a path-fixed and strictly uncontrollable mobile sink. We design a location-based greedy forwarding algorithm for *energy-optimal routing towards dynamic destination regions*, and demonstrate that the right-hand rule can also be used for void handling in this new context after being slightly modified.
2. An *effective location calibration method* is presented, which can be combined with the rough estimation based on the mobility pattern to determine the sink location. In this manner, the routing performance can be guaranteed with very low control overhead.
3. We propose an approach named *track routing* to deal with the sink location errors caused by delayed calibration or unpredicted faults. It can guarantee reliable and on-time delivery in an energy-efficient manner, by adopting a “greedily advance, discreetly step back” strategy.
4. We verify the effectiveness of our proposed method by extensive experiments and comprehensive performance comparisons. Additionally, we present a delay-constrained rendezvous-based routing, providing a supplementary baseline.

The remainder of this paper is organized as follows: Section 2 investigates the related work. Section 3 presents the network model and problem description. Section 4 elaborates the design of DERM. The performance evaluation is provided in Section 5. Finally, Section 6 concludes the paper.

## 2. Related Work

This paper mainly involves research from two aspects: data collection with mobile sinks, and geographic routing. We review the related work as follows.

### 2.1. Data Collection with Mobile Sinks

The issue of data collection in WSNs with mobile sinks has drawn much attention recently. Existing works are surveyed in [8,9], and can be summarized as three categories according to the sink mobility. As introduced in Section 1, they adopt different routing schemes, i.e., single-hop routing, multi-hop routing, and rendezvous-based routing.

*Random Mobility*: In [10], mobile entities are exploited to gather data via one-hop communication. The authors mathematically analyze the performance (e.g., energy efficiency, latency, and delivery ratio) based on the random mobility model. Li et al. present λ-flooding to achieve real-time data collection via multiple hops [11]. λ-flooding locally updates the collection tree and thus can effectively reduce the route maintenance overhead. In [12], the Predictive QoS Routing incorporates information potentials with the mobility graph to support data delivery in non-local sink movement scenarios. The mobility graph can be learned from training data and used to predict the future relay node nearest to the mobile sink. Lee et al. propose delivering data to stashing nodes along all predicted possible trajectories, and then the stashed data will be collected when the sink passes by [13].

*Path-Controllable Mobility*: Under this mobility pattern, many works are dedicated to designing the sink path for improving the network performance [15,16,17,18,19,20,21,22,23,24,25,26]. In [17], mobile elements visit each node to collect the buffered data using one-hop communication. The movements are scheduled to guarantee that the data can be delivered before buffer overflow. For multi-hop routing towards mobile sinks, several works [18,19,20] optimize the sink path to achieve energy balance in the network. A rendezvous-based approach [21,22] is introduced, with which data packets are first transferred to nodes along the scheduled path and further uploaded to a mobile collector when it arrives. Xing et al. aim to find the optimal path that minimizes the total route length and can be toured within the delay constraint. The work in [23] guarantees that all packets can be relayed to the mobile collector within bounded hops while the moving path length is minimized. The authors propose both centralized and distributed algorithms to determine the path. Xu et al. design the sink path so that the energy reduction will be maximized and a round-trip data collection can be completed within a specified time period [24]. In [25], a continuous sub-path on a given constrained trajectory is selected for the mobile sink to collect the maximum amount of data within the delay bound. Sha et al. find the moving paths for multiple sinks to achieve low-latency energy-efficient data gathering while exploiting the sleep scheduling and the sensing radius adjustment to reduce coverage redundancy [26].

*Path-Fixed Mobility*: The works in [27,28] optimize the energy consumption in WSNs with path-fixed mobile collectors. Several other proposals focus on the data collection on a pre-defined path in energy harvesting sensor networks. In [29,30], the amount of collected data is maximized by optimizing the time slot allocation to individual nodes along the path while their energy budget constraints can be satisfied. However, the above research assumes that all sensor nodes can directly communicate with the collectors in one hop, which might be infeasible in many scenarios. Luo et al. present a protocol named MobiRoute in which the mobile sink pauses at different anchor points along a fixed path and sensors transmit data packets to it through multiple hops [31]. To maximize the network lifetime, a 2-phase algorithm is used to allocate the pause time at each anchor point. In [32], nodes transfer data to corresponding cluster heads via the shortest path. When collecting data from these cluster heads within the direct communication range, the mobile sink can adjust its speed to accommodate the network conditions in different regions, and thus improve the network performance. Nevertheless, in some situations, the motion is strictly uncontrollable. Considering path-fixed mobile sinks with a constant speed, Gao et al. [33] suggest that optimizing the assignment of sensor nodes to subsinks (i.e., rendezvous nodes) can improve the network throughput as well as conserve the routing cost. This Maximum Amount Shortest Path (MASP) problem is solved with a genetic algorithm. However, it adopts the rendezvous-based routing mode, and does not take the delay performance into account. This paper complements the design of delay-aware energy-efficient data collection on a fixed path with a strictly uncontrollable mobile sink.

### 2.2. Geographic Routing

Geographic routing (also called position-based routing or location-based routing) is a long-known data forwarding strategy and has been widely studied [37]. It exploits the geographic information of one-hop neighbors to choose an optimal forwarder at each hop, and thus moves a packet to reach the destination node gradually. To ensure the QoS (Quality of Service) provisioning of geo-routing in WSNs, several routing metrics are introduced for optimizing the forwarder selection [38,39,40]. Greedy forwarding in geo-routing may fail when encountering a communications void (i.e., local minimum), and thus an effective approach is required to solve this problem for guaranteeing successful delivery. Existing void handling techniques are systematically surveyed in [41]. The authors classify them into six categories: planar graph-based, geometric, flooding-based, cost-based, heuristic, and hybrid. Therein, the face routing [42] and the perimeter routing in GPSR (Greedy Perimeter Stateless Routing) protocol [43], two very similar approaches, have become standard techniques for void handling. Unfortunately, above traditional geo-routing approaches cannot be directly applied for energy-optimal routing towards dynamic destination regions. Additionally, the problem of dealing with the sink location errors for reliable delivery still remains to be solved.

## 3. Network Model and Problem Description

In this section, we introduce the network model and relevant assumptions. Then, we describe the problem to be addressed in this work, and propose our basic approach.

### 3.1. Network Model

We consider a WSN with a mobile sink *M* and *N* static sensor nodes deployed in a monitoring area, as shown in Figure 1. Each node has the same transmission radius *R*. The sink periodically travels along a fixed path with a constant speed $v_{M}$, and knows its own mobility pattern, which is uncontrollable.

The sink path can be represented by a sequence of endpoints and turning points:

(1) $$P=(P_{0},P_{1},\;P_{2},...,P_{n}).$$

Note that, if *P* is a round-trip path, the points sequentially accessed by the sink in one cycle are $P_{0},P_{1},...,P_{n},...,P_{1},P_{0}$. If *P* is a cyclic path, those would be $P_{0},P_{1},...,P_{n},P_{0}$. We use tp to denote the path type (i.e., round-trip or cyclic). Therefore, the sink mobility pattern including the sink path, the speed, and the path type can be characterized by a triple of (*P*, $v_{M}$, tp). Let Ω(P) denote the direct communication region where the nodes’ distances to the sink path *P* are less than the communication radius *R*. Those nodes belonging to Ω(P) are called *potential rendezvous nodes* (RNs for short). We assume that each RN has enough storage space for buffering the received data. This is feasible as the development of the memory chip technology [33].

When the sink is moving, it sends out Beacon messages in regular intervals to discover nearby nodes. Each node receiving the message updates the Beacon timestamp in its sink list. If the timestamp has not been updated for a period of time, the sink will be regarded as out of the communication range.

Some other related assumptions are listed as follows:

1. Location awareness: All nodes know their physical locations and exchange them with their neighboring nodes in the initial phase. The locations can be obtained by GPS modules outdoors. In indoor environments, many existing methods (e.g., range-based and fingerprint-based techniques) can also achieve satisfactory localization accuracy [44].
2. Unreliable links: The wireless communication is unreliable due to channel fading, interference, etc. The MAC (Media Access Control) layer measures the link quality by calculating the Packet Reception Ratio (PRR) [45]. To ensure the reliability of one-hop packet delivery, the ARQ (Automatic Repeat reQuest) mechanism is adopted, by which a packet encountering transmission failures will be retransmitted until being acknowledged. For instance, in CC2530, the receiver can acknowledge a packet with the software ACK [46].
3. Time synchronization: The clocks of sensor nodes are synchronized, which can be achieved by GPS modules or a practical time synchronization method in WSNs such as FTSP (Flooding Time Synchronization Protocol) [47] or Glossy [48].
4. Data transmission time: The wireless signal travels much faster than the mobile sink. Therefore, compared to the delay constraint and the travel time of the sink, the time for multi-hop transmissions and data uploading from RNs to the sink is negligible [14]. Furthermore, when the RN finds that its cached data cannot be delivered within the communication time with the sink, it will transfer the excess data to a delegation node that can be visited before the deadline (see Section 4.2).

### 3.2. Problem Description

We assume a typical scenario that all sensor nodes generate packets randomly with a constant rate and the packets should be delivered to the sink before their own deadlines. Take the source node *s* in Figure 1 as an example. Our object is to find a delay-constrained energy-optimal route towards the mobile sink *M* for data packets generated from *s*. The end-to-end delay constraint is $D_{s}$. As mentioned before, we propose to first relay the packets to a node in the destination region via the shortest path. That node is called *destination node*, denoted by $N_{D}$. Then, the sink will pick up the cached data when it arrives within the time limit. The problem can be formulated as follows:

(2) $$\mathrm{minimize}\;\sum_{e\in Path(s,N_{D})}ETX(e),$$

Subject to

(3) $$\left\{\begin{array}{c} N_{D}\in \Omega \left(P\right),\\ t_{M\to N_{D}}\leq D_{s}. \end{array}\right.$$

Object function (2) minimizes the energy cost taking for transferring a packet from the source node *s* to the destination node $N_{D}$. The cost is defined as the total number of transmissions and can be estimated by the accumulated ETX (Expected Transmission Count) of all links (termed *e*) along the path from *s* to $N_{D}$ (termed $Path(s,N_{D})$). Equation (3) indicates that $N_{D}$ is located in the destination region, which means $N_{D}$ must be an RN, and the time for the sink *M* to reach it is less than $D_{s}$. We can see that the destination region varies as the sink moves.

## 4. Main Design of DERM

In this section, we first present an overview of DERM and then introduce the detailed design of each key component in DERM.

### 4.1. Design Overview

As analyzed in Section 3.2, DERM can be abstracted into the energy-optimal dynamic destination region anycast problem named EO-DRA.

**Figure 2:**
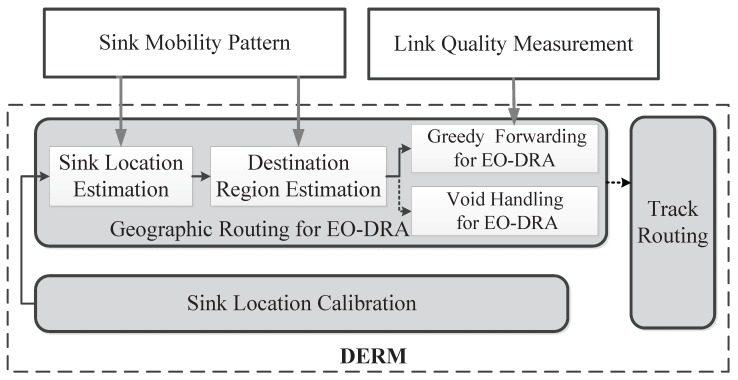
DERM framework overview.

To address this problem, the design of DERM mainly includes three components, as shown in Figure 2. Firstly, a *geographic routing protocol tailored for EO-DRA* is proposed. Each node estimates the current sink location and destination region by the sink mobility pattern. Then, it delivers the newly generating packet to any node in that region in a greedy manner based on a comprehensive metric considering both the location information and the link quality. To handle the communications void, we adapt the traditional perimeter routing for EO-DRA and demonstrate the efficacy. Secondly, we observe that the estimated location may not be accurate due to some factors such as pauses and velocity fluctuations. Therefore, an efficient *sink location calibration method* is adopted, with which each node updates the sink location when its route length dilation exceeds a certain limit caused by the outdated information. The method has low control overhead, and can still ensure high routing energy efficiency. Thirdly, for each node, there still exist unexpected sink location errors incurred by delayed updating or unpredicted movement deviations in future. Thus, we further propose a fault-tolerant mechanism named *track routing*. If a packet has been transferred to and buffered in an RN, but finds that the sink has not arrived by the deadline, it will exploit a reliable forwarding approach to track the sink along the path *P* and complete the last-mile delivery.   

To facilitate the subsequent descriptions of DERM, we introduce the network initialization operation here. In the initial phase, the sink broadcasts a message including its ID, mobility pattern (*P*, $v_{M}$, tp), initial location $P_{init}$, and the time to start moving $t_{init}$. Each node receiving the message stores this information in the sink list. All RNs will launch a procedure to preliminarily construct a route along the sink path, which will be elaborated in Section 4.4. Furthermore, each node exchanges its status information (i.e., node ID, PRR, and location) with the 1-hop neighboring nodes, and the graph planarization required for void handling is completed in a distributed manner.

### 4.2. Geographic Routing for EO-DRA

As is well known, geographic routing is an efficient routing scheme due to its scalability and localized feature, especially in the networks with dynamic topologies. However, unlike in the traditional geo-routing, in EO-DRA, the destination is a region determined by the sink path travelled within the delay constraint. Our target is to achieve energy efficient anycast towards the region, which makes the greedy forwarding and void handling in this context quite different.

#### 4.2.1. Greedy Forwarding for EO-DRA

We assume a packet originated from source node *s* is relayed to the current forwarding node *i*. Based on the mobility pattern (*P*, $v_{M}$, tp), the recorded sink location at a certain time, the node can roughly compute the current sink location and further estimate the travel path *L* within the delay constraint $D_{s}$. Note that the recorded location and its corresponding time are initialized with $P_{init}$ and $t_{init}$, respectively, and might be updated as introduced in Section 4.3.

Then, the node chooses a best forwarding node at the current time from its neighbors. Considering both link qualities and locations of nodes, we introduce a comprehensive routing metric named the remaining ETX (rETX), which can be estimated as:

(4) $$rETX_{ij}=ETX_{ij}+\left\lceil \frac{dist(j,L)}{\tilde{AvgPro(h_{i}+1)}}\right\rceil \cdot \tilde{AvgETX(h_{i}+1)},\;\left(h_{i}\geq 0\right),\;$$

where $rETX_{ij}$ represents the expected remaining ETX of the packet at node *i* when choosing neighboring node *j* as the next-hop forwarder. $ETX_{ij}$ can be calculated as the reciprocal of the PRR between nodes *i* and *j*. dist(j,L) is the shortest distance from node *j* to the polyline *L*. $h_{i}$ is the hop count from the source to the current node *i*. $\tilde{AvgPro(h_{i}+1)}$ and $\tilde{AvgETX(h_{i}+1)}$ denote the estimated average single-hop packet progress and ETX of the first $h_{i}+1$ hops, respectively. The single-hop packet progress (SPP) towards *L* when the packet is forwarded from *i* to *j* is defined as

(5) $$Pro_{ij}=dist\left(i,L\right)-dist\left(j,L\right).$$

We first calculate the actual average SPP and ETX of the elapsed $h_{i}$ hops [40]:

(6) $$AvgPro\left(h_{i}\right)=\frac{\left(h_{i}-1\right)\cdot AvgPro\left(h_{i}-1\right)+Pro\left(h_{i}\right)}{h_{i}},\;\left(h_{i}\geq 1\right),$$

(7) $$AvgETX\left(h_{i}\right)=\frac{\left(h_{i}-1\right)\cdot AvgETX\left(h_{i}-1\right)+ETX\left(h_{i}\right)}{h_{i}},\;\left(h_{i}\geq 1\right),$$

where $Pro(h_{i})$ and $ETX(h_{i})$ represent the actual SPP and ETX of the $h_{i}th$ hop forwarding, respectively. AvgPro(0) and AvgETX(0) at the source node can be set as zero because they are useless in our computation. Then, we have

(8) $$\tilde{AvgPro(h_{i}+1)}=\frac{h_{i}\cdot AvgPro\left(h_{i}\right)+\tilde{Pro(h_{i}+1)}}{h_{i}+1},\;\left(h_{i}\geq 0\right),$$

(9) $$\tilde{AvgETX(h_{i}+1)}=\frac{h_{i}\cdot AvgETX\left(h_{i}\right)+\tilde{ETX(h_{i}+1)}}{h_{i}+1},\;\left(h_{i}\geq 0\right),$$

where $\tilde{Pro(h_{i}+1)}$ and $\tilde{ETX(h_{i}+1)}$ are the expected SPP and ETX of the $(h_{i}+1)th$ hop forwarding, and can be estimated as the average of SPPs and ETXs of all *i*’s neighbors with a positive progress, respectively.

Therefore, we deduce the best forwarder *j* satisfying following conditions:

(10) $$\left\{\begin{array}{c} \underset{j\in N(i)}{min}\;\;rETX_{ij},\\ s.t.\;\;Pro_{ij}>0, \end{array}\right.$$

where N(i) denotes the neighboring node set of *i*. Based on the new metric, the packet will be forwarded towards the destination region in an efficient way.

If the packet arrives at an RN located in the destination region, it will be buffered until being collected by the sink. Moreover, if the RN finds that its buffered data cannot be uploaded to the mobile sink within the limited communication time, it will delegate the excess data to a neighboring RN that has spare communication time and can be visited within the delay constraint. If no such an RN can be found, the data will be transferred along the adjacent route (constructed in Section 4.4) to seek a delegation node.

**Figure 3:**
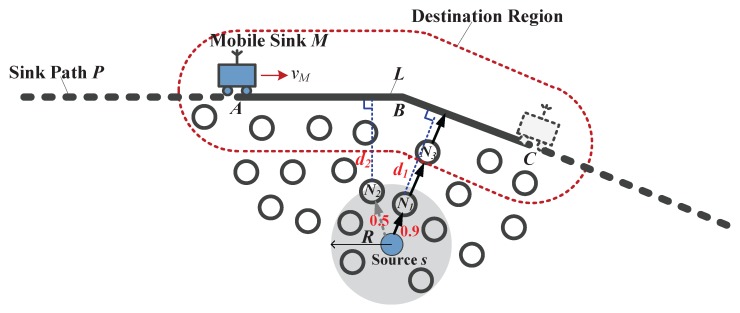
An example of greedy forwarding for EO-DRA.

We take Figure 3 as an example to illustrate the forwarding process. The source node *s* has a data packet required to be delivered within the delay constraint $D_{s}$. It looks up the target sink in its sink list, and estimates that *M* will travel from current location *A* to location *C* within the delay constraint. Thus, the travel path *L* is polyline (A,B,C). Node *s* calculates the rETX values for all its neighboring nodes having a positive SPP and finally chooses node $N_{1}$ as the next-hop forwarder. Note that node $N_{2}$ is closer to *L* than node $N_{1}$ ($d_{2}<d_{1}$), but it has a lower PRR (indicated near the edges) that incurs a larger rETX (i.e., more energy cost). Node $N_{1}$ receiving the packet will recalculate the destination region because the sink location might be updated, and select a forwarder as at the source. Unlike the traditional geo-routing where the destination location is recorded in the packet, we encapsulate the sink ID, along with the hop count *h*, the average SPP AvgPro(h), and the average ETX AvgETX(h). When the packet is relayed to node $N_{3}$, it will be cached until the sink arrives.

#### 4.2.2. Void Handling for EO-DRA

Greedy forwarding for EO-DRA may also encounter the communications void problem that a node (termed *void node*) fails to locate a next-hop forwarder closer to the destination region than it. The long-known perimeter routing [43] has been proved to be an efficient way to bypass the void area. However, it cannot be directly applied in DERM. We make the following modifications to adapt it for DERM: (1) the physical destination node is undetermined in advance unlike in traditional geo-routing. Thus, we define the *virtual destination* of a node as the point closest to the node on the sink’s travel path within the delay constraint. When a packet enters the void handling mode, it will be forwarded along the faces intersected by the line connecting the void node and its virtual destination using the right-hand rule; (2) we set the traversal direction (clockwise or counterclockwise) to be the same as the direction in which the void node, the current sink location, and the virtual destination can be visited orderly. In this way, the destination node will be closer to the sink, and the waiting time for the final delivery will be reduced.

**Figure 4:**
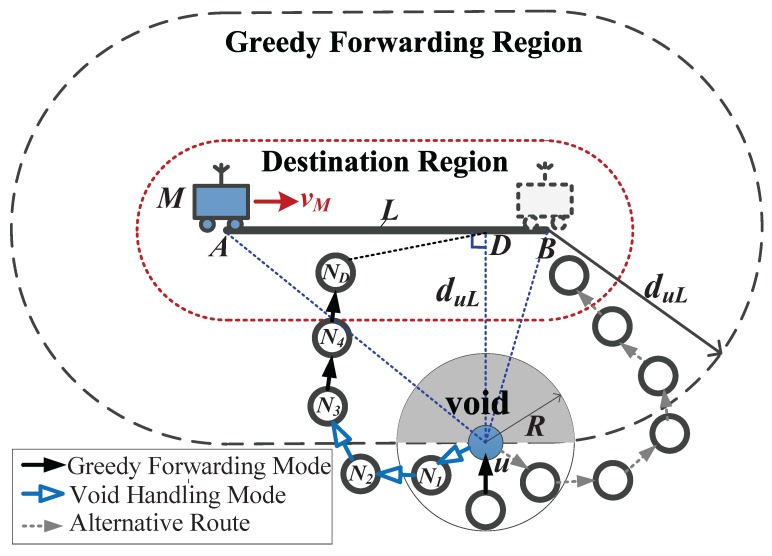
An example of void handling for EO-DRA.

As shown in Figure 4, a packet gets stuck at void node *u*. The shortest distance from *u* to the travel path *L* is $d_{uL}$. *u* cannot find a one-hop neighbor in the greedy forwarding region where the nodes’ distances to *L* are less than $d_{uL}$. Then, the stuck packet enters the void handling mode. Its virtual destination is *D* and the sink location is *A*. Points *u*, *A*, and *D* are sequentially visited in the clockwise direction. To find a path towards *L* clockwise, the packet traverses the faces crossed by the line $\bar{uD}$ using the counterclockwise direction of the right-hand rule. It will return to the greedy forwarding mode at node $N_{3}$.

It can be observed that the greedy destination region in EO-DRA is larger compared to the traditional geo-routing, which means that a packet suffers the communications void with a lower probability and can return to the greedy mode more easily.

We prove the efficacy of the modified perimeter routing for EO-DRA.

**Theorem** **1.** The modified perimeter routing for EO-DRA guarantees that a route from the void node to the destination region can always be found if it does exist.

**Proof.**  Assume that there exists a route from a void node *u* to the destination node $N_{D}$, $Path(u,N_{D})$. We add a virtual link connecting $N_{D}$ and the virtual destination *D* named $l(N_{D},D)$, and thus a route from *u* to *D* named Path(u,D) is formed. We have

(11) $$Path\left(u,D\right)=Path\left(u,N_{D}\right)\cup l\left(N_{D},D\right).$$

Based on the principle of the right-hand rule, travelling along the faces intersected by $\bar{uD}$ can find Path(u,D). $Path(u,N_{D})$ is a part of Path(u,D), and thus $N_{D}$ will be reached when travelling towards *D*. Figure 4 also illustrates this proof. ☐

### 4.3. Sink Location Calibration

As stated above, the sink location estimated by the mobility pattern may deviate from the real one, which will lead to inefficient routing. A straightforward solution is that the sink regularly broadcasts its location to the entire network. However, it will bring tremendous control overhead. Therefore, we introduce a location calibration method with which the updated location information is forwarded by a node (e.g., node *i*) only when the route length is enlarged beyond a threshold due to the stale information [11].

Let $d_{updt}$ denote the estimated routing path length of a packet when the updated sink location is used for forwarding, and let $d_{prev}$ denote that when the previous location information before updating is used. The method ensures $d_{prev}/d_{updt}\leq \alpha$, where α is the route length dilation threshold and α>1. $d_{updt}$ and $d_{prev}$ are estimated as

(12) $$d_{updt}=dist\left(i,L_{updt}\right),$$

(13) $$d_{prev}=dist\left(i,L_{prev}\right)+dist\left(D_{prev},L_{updt}\right).$$

$d_{updt}$ is calculated as the shortest distance from node *i* to the travel path within the delay constraint, termed $L_{updt}$, which is predicted based on the updated sink location. To compute $d_{prev}$, we first calculate the distance from *i* to the travel path $L_{prev}$ predicted based on the previous location, and obtain the corresponding virtual destination $D_{prev}$. If $D_{prev}$ is not on the path $L_{updt}$, to ensure on-time delivery, the packet should be further transferred to $L_{updt}$ via the track routing introduced in Section 4.4. Thus, $d_{prev}$ includes an additional item, $dist(D_{prev},L_{updt})$.

**Figure 5:**
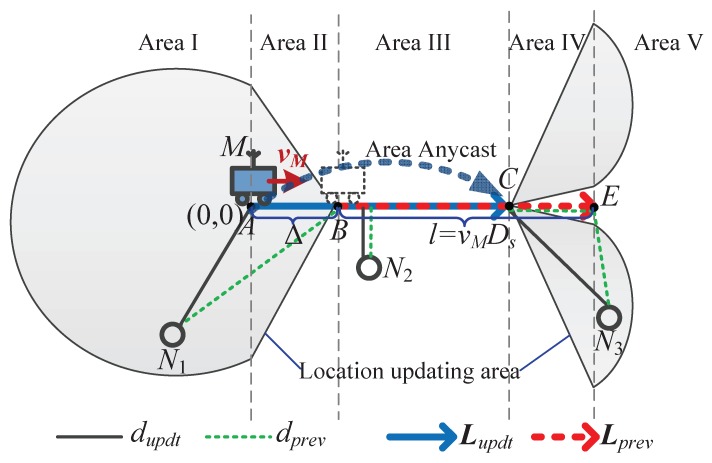
An example of the sink location calibration in DERM.

Figure 5 shows an example of the sink location calibration in DERM. The sink is currently located at *A* deviated from the estimated location *B* for Δ meters. The travel path length within the delay constraint is *l*. The sink broadcasts its current location information. Each node receiving the updated information will calculate its $d_{updt}$ and $d_{prev}$ based on Equations (12) and (13). Figure 5 depicts $d_{updt}$ and $d_{prev}$ of three nodes, i.e., $N_{1}$ in Area I, $N_{2}$ in Area III, and $N_{3}$ in Area V. Take node $N_{3}$ as an example. Its $d_{updt}$ is $|N_{3}C|$ and $d_{prev}$ is $|N_{3}E|+|CE|$. If $d_{prev}/d_{updt}>\alpha$, the node will update the sink location and forward it. Without loss of generality, the coordinate of *A* is set as (0, 0). A node whose coordinate is (x,y) will be required to forward the updated location if satisfying the following conditions:

(14) (x-Δ)2+y2(x-Δ)2+y2(x+y)2(x+y)2>α,(AreaI:x≤0),(x-Δ)2+y2(x-Δ)2+y2|y||y|>α,(AreaII:0<x≤Δ),(x-l+|y|)(x-l+|y|)(x-l)2+y2(x-l)2+y2>α,(AreaIV:l<x≤l+Δ),((x-l-Δ)2+y2+Δ)(x-l-Δ)2+y2+Δ(x-l)2+y2(x-l)2+y2>α,(AreaV:x>l+Δ).

From Equation (14), we can sketch out the location updating area (e.g., the shaded area in Figure 5).

It can be observed that: (1) exploiting the mobility for delay-aware routing can effectively reduce the overhead of location calibration, compared to the immediate multi-hop routing. For instance, nodes in Area III need not update the sink location as their routing paths are not affected by the estimation error; (2) the updated area is bounded, and its size is related to Δ and α. Only if the deviation Δ does exist and incurs route dilation at a node beyond the threshold α, the updating information will be relayed. These two facts indicate that the introduced method can deal with the sink movement deviations and guarantee energy-efficient routing in DERM at a very low cost.

The detailed procedure of the sink location calibration is shown in Algorithm 1. Node *i* records the updated sink location $P_{uM}^{i}$ and its last forwarded sink location $P_{lM}^{i}$. Each location entry has a corresponding time (e.g., $t_{uM}^{i}$ for $P_{uM}^{i}$). All nodes in the network set a location check timer. When the timer expires, the sink checks whether the deviation between its real location $P_{uM}$ and the estimated one $P_{eM}$ (deduced from $P_{lM}$) is beyond a threshold $\Delta_{th}$. If so, it broadcasts a location updating message LocMsg including $P_{uM}$ and the corresponding time $t_{uM}$ to its neighboring nodes (Lines 1–4). It is noted that the updating area might be separated into two parts as shown in Figure 5. Thus, the sink needs to send an area anycast message AnyCastMsg to a circular region [38] whose center point is $\tilde{P_{uM}}$ (the estimated sink location after $D_{s}$ seconds, e.g., point *C* in Figure 5) and radius is *R* (Line 5). If there are no nodes in that area, the radius will be increased and the anycast message will be retransmitted. When a node inside the target area receives the AnyCastMsg, as a new source of the updated information, it also floods a LocMsg to its neighbors (Lines 15–21). While receiving a new LocMsg, node *i* updates its previously recorded sink location $P_{uM}^{i}$ to $P_{uM}$. If $d_{prev}/d_{updt}>\alpha$, it propagates LocMsg to the neighborhood (Lines 22–28). Otherwise, it suppresses the forwarding of LocMsg temporally. Regularly, when the timer expires, the node checks $P_{lM}^{i}$, which is regarded as the previous location in this case. If $d_{prev}/d_{updt}>\alpha$, it broadcasts a LocMsg containing the updated information (Lines 9–12). The approach is obviously efficient because the sink does not need to send a message continuously to trigger the updating process when it moves.

### 4.4. Track Routing

As introduced in Section 4.1, the sink floods an announcement message to the entire network in the initial phase. In the meantime, the shortest path tree can be constructed as in [49]. Then, along the tree, each RN replies to the sink with a message including its location. Here, we define the *access point* of a node as the closest point to it on the sink path, which can be intuitively regarded as the site where the sink collects the data buffered in the node. We call two nodes “adjacent” if their access points are nearest in either direction along the path. The sink can find the adjacent nodes of each RN and send their locations to that RN through the corresponding reverse route. In this way, every RN is aware of its adjacent nodes, and thus a route sequentially connecting all the RNs along the path, called *adjacent route*, is established. Note that a route to an adjacent node outside the communication range can be discovered via geo-routing.

**Algorithm 1** Sink location calibration algorithm at sensor node *i*.**Input:**
α, $\Delta_{th}$; $P_{uM}^{i}$, $P_{lM}^{i}$, and their corresponding time; the sink mobility pattern;**Output:** newly calibrated sink location;1:**while** Location check timer expires **do**2:  **if**
*i* is the sink **then**3:    **if**
$dist\left(P_{uM},P_{eM}\right)>\Delta_{th}$
**then**4:      Broadcast $LocMsg(P_{uM},t_{uM})$ to its neighbors;5:      Send an $AnyCastMsg(\tilde{P_{uM}},R,P_{uM},t_{uM})$;6:      $P_{lM}\leftarrow P_{uM}$;7:    **end if**8:  **else**9:    **if**
$P_{uM}^{i}!=P_{lM}^{i}$ and $d_{prev}/d_{updt}>\alpha$
**then**10:      Broadcast $LocMsg(P_{uM}^{i},t_{uM}^{i})$ to its neighbors;11:      $P_{lM}^{i}\leftarrow P_{uM}^{i}$;12:    **end if**13:  **end if**14:**end while**15:**while** Receiving a new $AnyCastMsg(\tilde{P_{uM}},R,P_{uM},t_{uM})$
**do**16:  **if**
*i* is inside the target area **then**17:    Broadcast $LocMsg(P_{uM},t_{uM})$ to its neighbors;18:  **else**19:    Forward the AnyCastMsg greedily;20:  **end if**21:**end while**22:**while** Receiving a new $LocMsg(P_{uM},t_{uM})$
**do**23:  $P_{uM}^{i}\leftarrow P_{uM}$;24:  **if**
$d_{prev}/d_{updt}>\alpha$
**then**25:    Forward $LocMsg(P_{uM},t_{uM})$ to its neighbors;26:    $P_{lM}^{i}\leftarrow P_{uM}$;27:  **end if**28:**end while**

When a packet finds that the sink has not visited the RN it buffered in before the deadline due to delayed location updating or unpredicted faults, it must be delivered via multi-hop routing. To address this problem, we propose a method named track routing, as shown in Algorithm 2. The design of Algorithm 2 is elaborated as follows.

#### 4.4.1. Determining the Forwarding Direction

**Figure 6:**
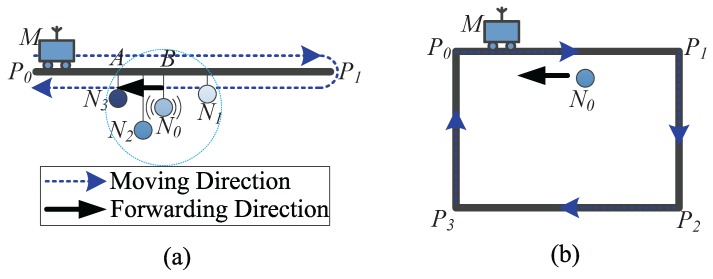
Examples of determining the forwarding directions for (**a**) the round-trip path; and (**b**) the cyclic path.

When a packet enters the track routing mode at a node called *tracking node* (e.g., $N_{0}$ in Figure 6), it will determine the packet forwarding direction first (Lines 2–20). Our method supports two types of sink paths, i.e., the round-trip path and the cyclic path.

For the round-trip path, the tracking node broadcasts a message to its neighbors to query their sink Beacon timestamps. Obviously, the fresher the timestamp, the closer the distance between a node and the sink. Therefore, the neighboring RN having the freshest timestamp will be selected as the next-hop forwarder. Assuming the timestamps of the tracking node and node *i* are $t_{0}$ and $t_{i}$, respectively, the forwarder has a maximum $t_{i}-t_{0}$. If there are no RNs in the neighborhood, the tracking node will choose the adjacent node with a fresher timestamp as the forwarder. The packet forwarding direction (termed fwdDirec) can be represented by a vector from the current node to the next-hop node. For example, in Figure 6a, $N_{3}$ is the forwarder and the vector $\vec{N_{0}N_{3}}$ (or $\vec{BA}$) indicates fwdDirec.

**Algorithm 2** Track routing algorithm at sensor node *i*.**Input:** the neighbor node set N(i); the sink path, the timestamp $t_{i}$; fwdDirec, reverse_flag;**Output:** the next-hop forwarder;1:**while** receiving or buffering a packet in the track routing mode **do**2:  **if** node *i* is the tracking node **then**3:    **if**
tp==round-trip
**then**4:      Find the node *j* with the maximum $t_{j}$ in N(i);5:      **if**
j!=null
**then**6:        Forward the packet to *j*;7:        $fwdDirec=\vec{i\;j}$;8:      **else**9:        Forward the packet to the adjacent node *k* with the maximum $t_{k}$;10:        $fwdDirec=\vec{i\;k}$;11:      **end if**12:    **else**13:      Determine fwdDirec based on Equation (15);14:      Find the node *j* satisfying Equation (17);15:      **if**
j!=null
**then**16:        Forward the packet to *j*;17:      **else**18:        Forward the packet to the adjacent node in fwdDirec;19:      **end if**20:    **end if**21:  **else**22:    **if**
dist(i,M)≤R
**then**23:      Deliver the packet to *M*;24:    **else if**
reverse_flag==true
**then**25:      Forward the packet to the adjacent node in fwdDirec;26:    **else if**
is_missed==true
**then**27:      Reverse fwdDirec and forward the packet to the adjacent node in new fwdDirec;28:      reverse_flag=true;29:    **else**30:      Find the node *j* satisfying Equation (17);31:      **if**
j!=null
**then**32:        Forward the packet to *j*;33:      **else**34:        Forward the packet to the adjacent node in fwdDirec;35:      **end if**36:    **end if**37:  **end if**38:**end while**

For the cyclic path, the sink travels along the path in a uniform direction all the time. Thus, moving the packet to chase the sink based on the freshness of timestamps may result in a long detour. In this case, fwdDirec is a Boolean value and can be set as:

(15) fwdDirec=same,if(tcurrent-t0)modT≤TT22,opposite,otherwise,

where $t_{current}$ denotes the current time and *T* is the time for the sink to complete one cycle of movement. If the time since the sink passed node $N_{0}$ is beyond T/2, it may have travelled half the path with respect to $N_{0}$. Hence, the packet will be forwarded in the opposite direction of the sink movement, as shown in Figure 6b.

#### 4.4.2. Selecting the Forwarder

After determining fwdDirec, a node would select a best next-hop forwarder (Lines 21–37). An intuitive solution is directly forwarding the packet to its adjacent node in the direction fwdDirec. However, it will make the packet visit the RNs one by one along the path towards the sink, which is obviously inefficient. We perform the track routing in a greedy manner.

To facilitate the presentation, we define $Adv_{fD}^{P}\left(i,j\right)$ as the advance along the path *P* in the direction fwdDirec when a packet is forwarded from node *i* to *j*. Denote $P_{i}$ and $P_{j}$ as the access points of *i* and *j* on the path *P*, respectively. $Path(P_{i},P_{j})$ represents the path from $P_{i}$ to $P_{j}$, which may include turning points of *P*. $Adv_{fD}^{P}\left(i,j\right)$ can be calculated as

(16) $$Adv_{fD}^{P}\left(i,j\right)=\left\{\begin{array}{c} ||Path\left(P_{i},P_{j}\right)|{|}_{2},\;\;if\;\mathrm{direc}\left(Path\left(P_{i},P_{j}\right)\;\right)=fwdDirec,\\ -||Path\left(P_{i},P_{j}\right)|{|}_{2},\;\;otherwise. \end{array}\right.$$

If $Path(P_{i},P_{j})$ has the same direction as fwdDirec, $Adv_{fD}^{P}\left(i,j\right)$ equals the length of $Path(P_{i},P_{j})$. Otherwise, it is negative.

When node *i* receives a packet in the track routing mode, it will first check whether the sink is within the communication range. If so, the packet will be directly delivered. Otherwise, *i* chooses a neighbor *j* as the forwarder which satisfies following conditions:

(17) $$\left\{\begin{array}{c} \max_{j\in N(i)}Adv_{fD}^{P}\left(i,j\right),\\ s.t.\;\;j\in \Omega (P),\\ 0<Adv_{fD}^{P}\left(i,j\right)<\beta R. \end{array}\right.$$

β is a factor used to restrict the single-hop advance because an excessively large advance may result in missing the mobile sink. Generally, we set β as 1. If *i* cannot find such a node *j*, it will forward the packet to its adjacent node in the direction fwdDirec.

We also consider an infrequent situation that the packet misses the sink due to a long advance. Assume the timestamps of node *i* and its last-hop node are $t_{i}$ and $t_{last}$, respectively. The situation will be recognized if (1) $t_{i}<t_{last}$ when the path is round-trip; or (2) $t_{i}<t_{last}$ when the path is cyclic and fwdDirec is same; or (3) $t_{i}>t_{last}$ when the path is cyclic and fwdDirec is opposite. The principle is that the Beacon timestamps mutate at the node being visited by the sink. In this case, the forwarding direction will be reversed. The packet will be forwarded back to the adjacent node in new fwdDirec and enter the reverse track routing mode. When receiving a packet in the reverse track routing mode, a node just transfers it to the adjacent node in fwdDirec.

**Figure 7:**
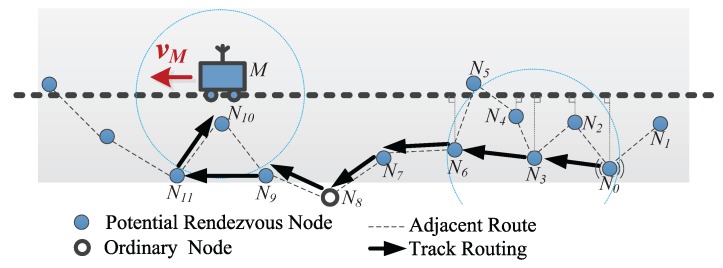
An example of the track routing in DERM.

We take Figure 7 as an example to illustrate the track routing process in DERM. The dashed lines represent the adjacent route. A packet at node $N_{0}$ needs to be delivered to the sink *M*. As in Figure 6a, the packet will be first transferred to $N_{3}$ with the freshest timestamp, and the forwarding direction is determined as $\vec{N_{0}N_{3}}$. Then, $N_{3}$ chooses $N_{6}$, which has the maximum $Adv_{fD}^{P}$ as the next-hop forwarder. Note here that forwarding to the adjacent node $N_{4}$ will incur a longer route. On receiving the packet, $N_{7}$ fails to find a neighboring RN and sends it to the adjacent node $N_{9}$ through an ordinary node $N_{8}$ outside the region Ω(P). When the packet arrives at $N_{11}$, it realizes that the sink is missed. $N_{11}$ will reverse the forwarding direction and transmit the packet to its adjacent node $N_{10}$, which is within the communication range of *M*.

It can be observed that the track routing adopts a “greedily advance, discreetly step back” strategy, achieving high energy efficiency as well as reliable data delivery. Compared to the pheromone-based forwarding scheme [14], with our approach, the packet will not get stuck when failing to locate a neighboring node with a fresher timestamp. It takes full advantage of the path information, and thus (1) just needs to query the timestamps once for determining the forwarding direction and (2) can also obtain better performance for the cyclic path.

## 5. Implementation and Performance Evaluation

In this section, we implement DERM and three other solutions in the ns-2 simulator [50], and compare their performance. The immediate multi-hop routing is used in [31] for sensor nodes to transmit data packets to the mobile sink when it pauses at anchor points. The rendezvous-based routing is adopted in [32], with which each node transfers data to an RN via the shortest path. We adapt these two approaches for WSNs with a path-fixed and strictly uncontrollable mobile sink. In addition, we present a delay-constrained rendezvous-based scheme as a supplementary baseline. Their routing strategies are introduced below.

- *Immediate Multi-hop Routing (Multihop)*: All nodes send data packets immediately to the mobile sink using geographic routing. The location of the sink is estimated and calibrated as in DERM, and the track routing mechanism is exploited to dealing with the location deviations.
- *Rendezvous-based Routing (Rendezvous)*: Each data packet is transmitted to an RN via the shortest path using geo-routing and then collected when the sink arrives.
- *Delay-Constrained Rendezvous-based Routing (DC-rendezvous)*: Each packet is first sent to the closest RN as in the traditional rendezvous-based routing. However, if the sink has not arrived within the delay constraint, the packet will be delivered to it via the track routing.

For fair comparisons, the aforementioned three solutions and DERM adopt the same routing metric (i.e., remaining ETX) as defined in Equation (4). We first evaluate their performance in ideal scenarios where the sink movement exactly complies with its mobility pattern. Then, we show the simulation results in realistic scenarios where the real movement deviates from the estimated one, and demonstrate the effectiveness of the void handling and the track routing mechanisms in DERM for reliable packet delivery.

### 5.1. Simulation Setup

We use the *setdest* tool to generate 100 different topologies randomly in a 1000 m × 400 m rectangular area with four vertices located at (0 m, 0 m), (0 m, 400 m), (1000 m, 400 m), and (1000 m, 0 m). The network consists of 1000 nodes and the communication ranges are set as 40 m. We adopt the IEEE 802.15.4 MAC layer protocol and measure the PRR with the Nakagami fading model [51] where the link quality is related to the distance between two nodes. Each node randomly generates one sensory data packet per minute, which is required to be delivered within the delay constraint. The mobile sink moves from the start point (0 m, 200 m) to the end point (1000 m, 200 m) and then returns back to the start point with a constant speed. It sends out a Beacon message every second when moving in the network. The sink location check timer interval is 5 s and the deviation threshold $\Delta_{th}$ is 1 m. Unless otherwise stated, we set the sink speed $v_{M}$, the delay constraint $D_{s}$, and α as 2 m/s, 120 s, and 1.2, respectively. All the results have been averaged over 100 rounds of simulations.

### 5.2. Evaluation Metrics

We evaluate the performance of our design in terms of the following metrics:

- *Transmission Cost per Packet*: measured as the average number of transmissions for an end-to-end (E2E) packet delivery.
- *E2E Delivery Delay per Packet*: the average elapsed time from a data packet being sent out by the source node to finally being collected by the mobile sink.
- *Maximum E2E Delivery Delay*: the maximum of end-to-end delivery delays of all packets generated at different sensor nodes.
- *Location Calibration Overhead*: measured as the average number of sink location updates per node during one cycle of sink movement.
- *Packet Delivery Success Ratio*: the ratio of the number of packets successfully received by the sink to the total number of packets sent from source nodes.
- *On-time Delivery Ratio*: the ratio of the number of packets delivered to the sink within the delay constraint to the total number of packets sent by source nodes.

### 5.3. Performance Evaluation in Ideal Movement Scenarios

In ideal scenarios, the sink location can be accurately estimated using the mobility pattern, and thus the location calibration mechanism is not required. We mainly investigate the impact of the sink speed and the delay constraint on the performance of DERM and the three baseline solutions.

#### 5.3.1. Performance Overview in Ideal Movement Scenarios

**Figure 8:**
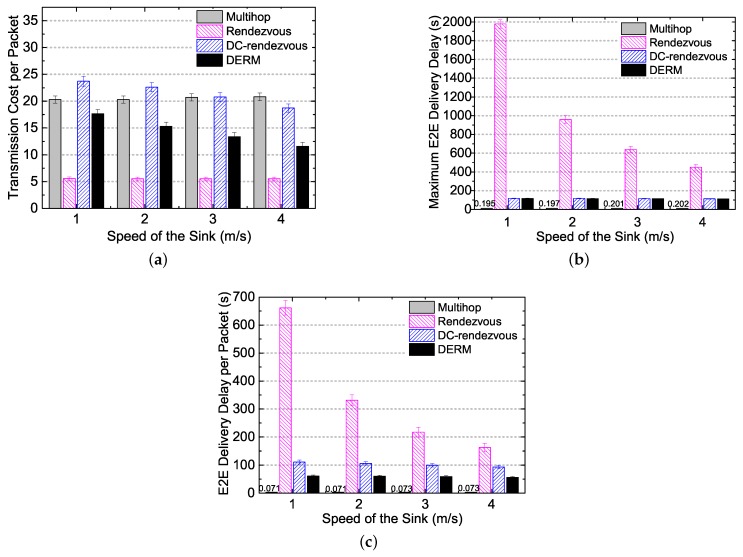
Performance overview under different sink speeds in ideal movement scenarios ($D_{s}$ = 120 s). (**a**) transmission cost per packet; (**b**) maximum end-to-end delivery delay; (**c**) end-to-end delivery delay per packet.

We evaluate the performance of DERM in ideal movement scenarios, varying the sink speed from 1 to 4 m/s. Figure 8a reports the transmission cost per packet of the four routing schemes under different sink speeds. Compared to the *Multihop* and the *DC-Rendezvous* approaches, DERM reduces the transmission cost by 13%–44% and 26%–38%, respectively. The energy savings are significant considering the cost is calculated by averaging the transmission number per node per packet. This is because DERM takes full advantage of the sink mobility. Although *DC-Rendezvous* also exploits the mobility to some extent, it does not utilize the sink location information for routing and thus achieves lower energy efficiency compared with DERM. As expected, the *Rendezvous* approach has the smallest transmission cost but at the price of tremendous delivery delay since it simply transmits packets to the vicinity of the sink path. As the sink speed increases, the transmission cost per packet of DERM and *DC-Rendezvous* decreases. The reason is that a higher speed leads to a larger destination region, which provides more room for obtaining an energy-efficient route.

Figure 8b shows that *Multihop* achieves real-time data transmission. DERM and *DC-Rendezvous* can also meet the delay requirement as their maximum E2E delivery delays are both no greater than 120 s. On the contrary, the maximum E2E delay of *Rendezvous* is uncontrollable, which all depends on the path length and the sink speed. Figure 8c indicates the results in terms of the E2E delivery delay per packet. *DC-Rendezvous* yields a larger E2E delay per packet than DERM. It can be explained by the fact that, in *DC-Rendezvous*, many packets wait for $D_{s}$ seconds and finally find that the sink has not arrived. The maximum E2E delay and E2E delay per packet of *Rendezvous* decrease significantly when the speed is increased from 1 to 4 m/s because the sink can reach a rendezvous node in less time with a higher speed. Regarding DERM and *DC-Rendezvous*, the maximum E2E delay almost stays unchanged and the E2E delay per packet decreases at a slow rate with increasing of the speed, due to the delay-aware operation.

#### 5.3.2. Impact of the Delay Constraint

**Figure 9:**
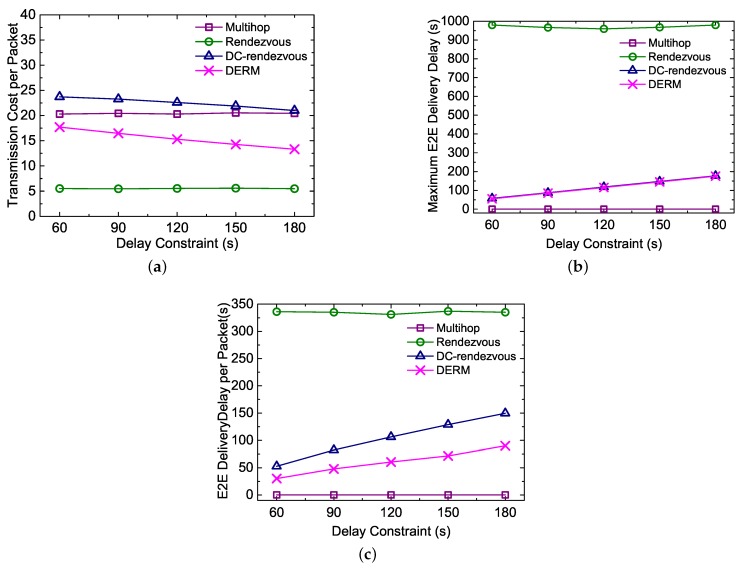
Impact of the delay constraint on the performance of four routing schemes ($v_{M}$ = 2 m/s). (**a**) transmission cost per packet; (**b**) maximum end-to-end delivery delay; (**c**) end-to-end delivery delay per packet.

In Figure 9, we study the impact of the delay constraint on the performance of different routing schemes. Figure 9a shows that the transmission cost per packet of DERM and *DC-Rendezvous* decreases by 25% and 11%, respectively, when the delay constraint is increased from 60 to 180 s. This is because, like increasing the speed, relaxing the delay constraint will also enlarge the destination region. Figure 9b illustrates that DERM as well as *DC-Rendezvous* can always meet the corresponding delay requirement when the constraint is changed. Not surprisingly, the performance of *Multihop* and *Rendezvous* is little affected by the delay constraint, as shown in Figure 9a–c.

### 5.4. Performance Evaluation in Realistic Movement Scenarios

In realistic scenarios, the real sink locations may deviate from the values estimated by the mobility pattern. To test the effectiveness of DERM in such movement scenarios, we assume that the mobile sink pauses at the midpoint of the path for a period of time. The pause time is set to 30 s by default.

#### 5.4.1. Performance Overview in Realistic Movement Scenarios

**Figure 10:**
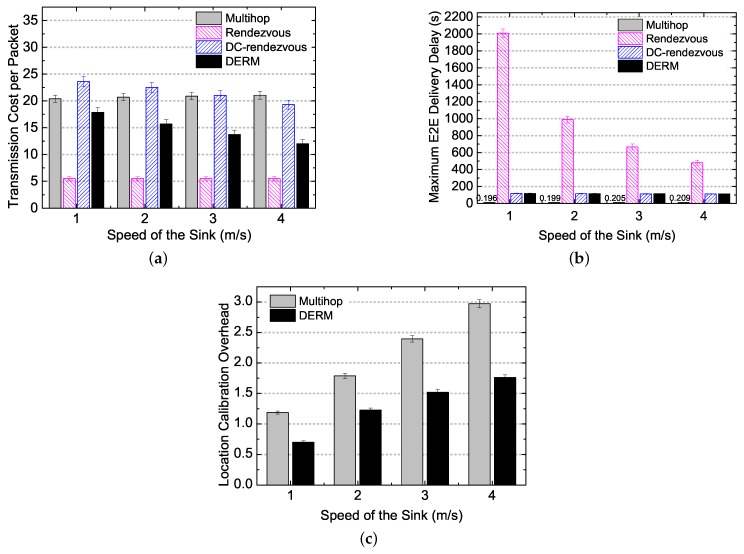
Performance overview under different sink speeds in realistic movement scenarios ($D_{s}$ = 120 s, α = 1.2, and the pause time is 30 s). (**a**) transmission cost per packet; (**b**) maximum end-to-end delivery delay; (**c**) location calibration overhead.

Figure 10 reports the evaluation results under different sink speeds in realistic movement scenarios. From Figure 10a,b, we can observe that the four schemes achieve very close transmission cost per packet and maximum E2E delays to those shown in Figure 8a,b, respectively. There are only some increases in the maximum E2E delay of *Rendezvous* (e.g., from 1979 to 2008 s when the speed is 1 m/s) due to the pause. This means that the location calibration method performs well in tackling the location deviations for *Multihop* and DERM. As for *Rendezvous* and *DC-Rendezvous*, the location information is not used. The transmission cost of DERM decreases by 13%–43% and 24%–38% compared to *Multihop* and *DC-Rendezvous*, respectively. Furthermore, the location estimation errors inevitably exist in realistic movement scenarios, but the maximum E2E delay of DERM can still meet the delay constraint, which demonstrates the efficiency of the track routing for ensuring on-time delivery. Figure 10c shows the control overhead of the sink location calibration. The average number of updates per node in *Multihop* and DERM increases from 1.2 to 3 and 0.7 to 1.8, respectively, when the speed is varied from 1 to 4 m/s. This is because the location deviation is proportional to the speed with a given pause time. Remarkably, compared to *Multihop*, the overhead of DERM is reduced by about 40% and increases at a smaller rate with the speed. For DERM, 0.7–1.8 updates per node during one cycle of the sink movement is acceptable.

#### 5.4.2. Impact of α

In this experiment, we investigate the impact of α on the performance of DERM and *Multihop*. For each approach, we show a lower bound (i.e., optimal value) of the transmission cost per packet, which can be achieved when the real sink movement is always known. We also present the upper bound, which is obtained when the location calibration method is not adopted.

**Figure 11:**
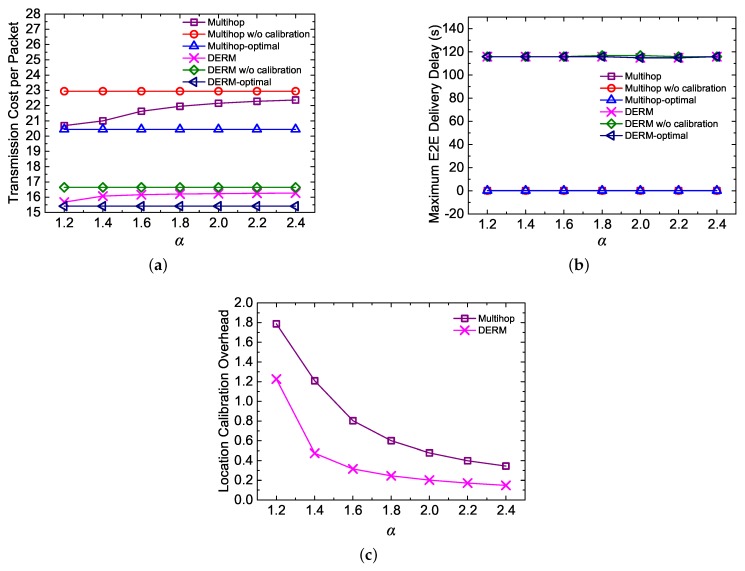
Impact of α ($v_{M}$ = 2 m/s, $D_{s}$ = 120 s, and the pause time is 30 s). (**a**) transmission cost per packet; (**b**) maximum end-to-end delivery delay; (**c**) location calibration overhead.

As depicted in Figure 11a, the transmission cost of DERM and *Multihop* increases with α since a greater route length dilation is allowed. Figure 11b shows that α has almost no influence over the maximum E2E delay because the track routing can always guarantee on-time packet delivery. On the other hand, the location calibration overhead is reduced when α is varied from 1.2 to 2.4, as shown in Figure 11c. We see that adjusting α can strike a balance between the routing efficiency and the overhead. It is noted that, when α is set to 1.2, the two approaches achieve approximately optimal transmission cost. For DERM and *Multihop*, the number of transmissions per packet is reduced by about 1 and 2.3, respectively, compared with their corresponding upper bounds. Therefore, under this setting, the reductions are considerable while the control overhead is acceptable.

#### 5.4.3. Impact of the Pause Time

**Figure 12:**
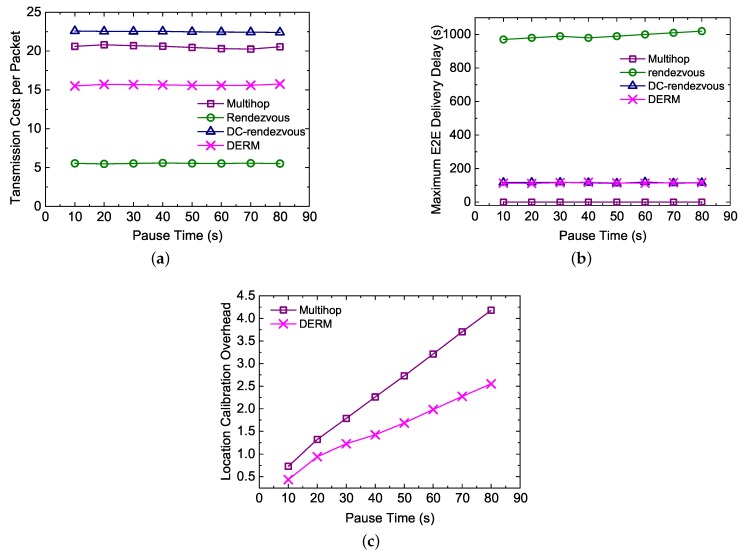
Impact of the pause time ($v_{M}$ = 2 m/s, $D_{s}$ = 120 s, and α = 1.2). (**a**) transmission cost per packet; (**b**) maximum end-to-end delivery delay; (**c**) location calibration overhead.

In this test, we examine the performance of the four routing schemes under different pause times varying from 10 to 80 s. Figure 12a indicates that the transmission cost of DERM and *Multihop* change little with the pause time, although longer pauses will incur larger location deviations. This result demonstrates the effectiveness of the location calibration method. Judging from Figure 12b, the maximum E2E delay of *Rendezvous* gradually increases with the pause time, while that of the other three approaches almost stays constant and satisfies the constraint. As expected, the location calibration overhead is proportional to the pause time, as shown in Figure 12c. Compared to *Multihop*, the overhead of DERM increases more slowly with the increase of the pause time, which implies that *DERM is more applicable for the realistic scenarios*.

#### 5.4.4. Effectiveness of DERM for Reliable Packet Delivery

**Figure 13:**
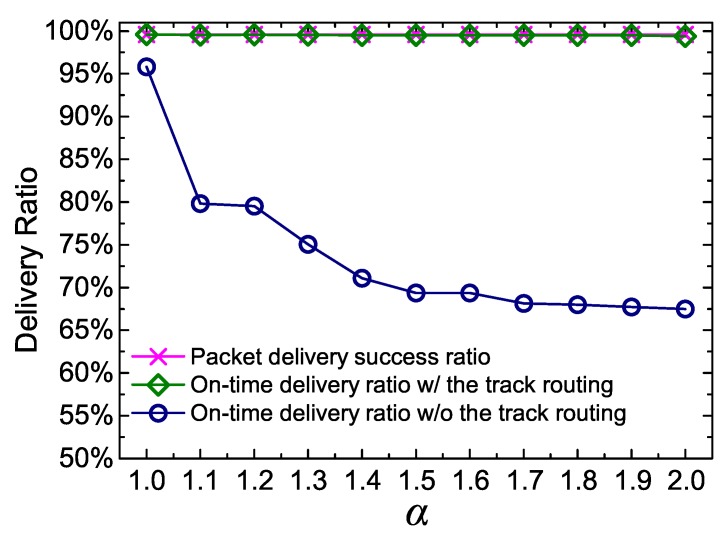
Delivery ratio of DERM with and without the track routing ($v_{M}$ = 2 m/s, $D_{s}$ = 120 s, and the pause time is 30 s).

In this part, we verify the effectiveness of DERM for packet successful delivery and on-time delivery. As illustrated in Figure 13, DERM achieves a high delivery success ratio close to 100%, which can be mainly attributed to the modified perimeter routing employed in DERM for handling communications void. Another reason is that the destination in DERM is a region, and the probability of having a route from a node to a region is higher than to a point. When the track routing is adopted, the on-time delivery ratio stays approximately the same as the success ratio despite the increase of α. That is, almost all the received packets are delivered within the delay constraint. For comparison purposes, we also present the on-time delivery ratio of DERM without the track routing. As α is varied from 1.0 to 2.0, the ratio decreases from 96% to 67% because more nodes suffer sink location errors, but no fault-tolerant mechanism is used. Note that, when α is set to 1, the on-time delivery still cannot be guaranteed without the track routing. This is because the future sink location deviations cannot be predicted, although the real current location is always known. From the above observations, we can see that the track routing is effective as well as necessary.

### 5.5. Performance Summary

From above observations, we can conclude that DERM achieves the optimal energy efficiency under the delay constraint. Compared with *Rendezvous* and *DC-rendezvous*, DERM needs to estimate the sink location and thus introduces the extra control overhead for location calibration in realistic movement scenarios. However, DERM has much better energy efficiency than *DC-rendezvous*, and can always meet the delay requirements compared to *Rendezvous*. The overhead can be amortized by energy savings on the delivery of continuously generated data packets. *Multihop* can achieve real-time packet delivery, but it also brings more transmission costs and requires more frequent location calibration compared to DERM.

## 6. Conclusions

In this work, we propose DERM, a protocol aiming at achieving delay-aware energy-optimal routing in WSNs with a path-fixed mobile sink. DERM employs a location-based greedy forwarding technique, enabling each node to relay packets to a destination region accessible to the mobile sink within the delay constraint. An energy-efficient location calibration method is introduced to deal with the deviation between the estimated and the real sink movements. Moreover, we present the track routing to guarantee packet on-time and reliable delivery. Extensive evaluation results demonstrate the effectiveness of our proposed approach in both ideal and realistic movement scenarios. Compared to *Multihop* and *DC-rendezvous*, DERM reduces the transmission cost by 13%–44% and 24%–38%, respectively, and requires about 40% less control overhead than *Multihop* for the sink location calibration. Compared with *Rendezvous*, it can always meet a wide range of delay constraints under different settings. The energy profit brought by DERM is proportional to the speed and the delay constraint. Therefore, DERM can be applied in many industrial applications with various delay requirements for energy conservation due to its scalability and flexibility.

In the future, we plan to validate the proposed approach in WSNs with multiple path-fixed sinks. Furthermore, we also plan to enhance our design by comprehensively considering the end-to-end delay, the transmission cost as well as the total amount of collected data.
